# Mobile App–Based Self-Management of Urinary Incontinence in Pregnant Women: Multicenter Pragmatic Randomized Controlled Trial

**DOI:** 10.2196/72883

**Published:** 2025-08-07

**Authors:** Yuling Zhu, Wenzhi Cai, Sha Liu, Danli Zhang, Dan Luo, Shaoli Huang, Wei Shi, Lihua Yang, Shuanghao Zhang, Hengying Fang, Ling Chen

**Affiliations:** 1 Department of Nursing, Shenzhen Hospital, Southern Medical University, Number 13, Xinhu Road, Bao’an District Shenzhen, Shenzhen, 518101, China, 86 19925360554; 2 School of Nursing, Southern Medical University, Guangzhou, China; 3 Department of Urology Surgery, Shenzhen Hospital, Southern Medical University, Shenzhen, China; 4 Department of General Surgery, Shenzhen Hospital, Southern Medical University, Shenzhen, China; 5 Department of Gastrointestinal Surgery, Shenzhen Hospital, Southern Medical University, Shenzhen, China; 6 Department of Obstetrics and Gynecology, Foshan Shunde District Third People's Hospital (Beijiao Hospital), Foshan, China; 7 Department of Pelvic Floor Rehabilitation Medicine, Qinghai Kangle Hospital, Xining, China; 8 Department of Obstetrics, Affiliated Hospital of Xiangnan University, Chenzhou, China; 9 Women's Health Center, Dongguan Maternal and Child Health Hospital, Dongguan, China; 10 Department of Nursing, The Third Affiliated Hospital of Sun Yat-sen University, Guangzhou, China

**Keywords:** urinary incontinence, mobile app, mHealth, pregnancy, pelvic floor muscle training, self-management, multicenter randomized controlled trial

## Abstract

**Background:**

Urinary incontinence (UI) is a common condition during pregnancy, significantly impacting the physical and mental well-being as well as quality of life. With advancements in mobile health technology, mobile apps provide innovative approaches for managing UI. Although small-scale studies have demonstrated their efficacy in alleviating maternal UI symptoms, there is a notable lack of large-scale, multicenter trials to validate these findings.

**Objective:**

This multicenter randomized controlled trial evaluated the effectiveness of a mobile app (“Urinary Incontinence for Women”) for self-management of UI among pregnant women.

**Methods:**

A total of 295 participants were recruited from obstetric clinics at 5 hospitals and randomized to either a 12-week mobile app–based intervention group (n=148) or a standard care group (n=147). The primary outcome was UI symptom severity, with secondary outcomes of the impact of UI on quality of life and self-efficacy in pelvic floor muscle training (PFMT). Assessments were conducted at baseline, postintervention (12 weeks), and 6‐8 weeks postpartum via electronic questionnaires. Generalized estimating equation modeling was used to evaluate the intervention effects. Subgroup analyses were performed to examine intervention effects across different baseline characteristics.

**Results:**

A total of 267 participants (267/295, 90.5%) completed all assessments. The intervention group showed significantly improved UI symptom severity compared with controls postintervention (β=−.97, 95% CI −1.85 to −0.08; *P*=.03), with further improvement at 6‐8 weeks postpartum (β=−1.93, 95% CI −2.70 to −1.17; *P*<.001). The impact of UI on quality of life also improved in the intervention group postintervention (β=−1.05, 95% CI −1.95 to −0.15; *P*=.048) and at 6‐8 weeks postpartum (β=−1.72, 95% CI −2.46 to −0.98; *P*<.001). No significant between-group difference was observed in PFMT self-efficacy (β=1.49, 95% CI −2.93 to 5.90; *P*=.51), which decreased significantly from postintervention to 6‐8 weeks postpartum (β=−4.66, 95% CI −8.11 to −1.22; *P*=.008). Subgroup analyses revealed significant interactions between intervention effects and education level, PFMT performing before enrollment, history of vaginal delivery, and baseline UI symptoms.

**Conclusions:**

The “Urinary Incontinence for Women” app–based self-management strategies significantly improved UI symptom severity and quality of life in pregnant and postpartum women, with pronounced effects in certain subgroups based on education level and baseline UI status. While PFMT self-efficacy was not enhanced, the app’s benefits underscore the clinical relevance of personalized UI management.

## Introduction

Urinary incontinence (UI), defined by the International Continence Society as the involuntary leakage of urine through the urethra [1], represents a significant health concern that substantially impacts women’s physical, psychological, and social well-being [2,3]. Women experiencing UI often face emotional challenges [4-6], including anxiety, depression, and low self-esteem, alongside social embarrassment and sexual dysfunction. These complications, coupled with considerable economic burdens [7,8], underscore UI as a major public health issue.

The physiological changes during pregnancy and childbirth significantly alter pelvic floor function, making pregnant women particularly vulnerable to UI [9]. Studies report UI prevalence rates ranging from 33.4% to 62.7% across different trimesters, with an increasing trend as pregnancy progresses [10-13]. More concerning is that UI during pregnancy often persists beyond the gestational period, serving as a predictor for postpartum UI and long-term pelvic floor dysfunction [14,15]. This trajectory emphasizes the critical importance of preventive interventions beginning in early pregnancy.

Pelvic floor muscle training (PFMT) is considered the gold standard for conservative UI management [16], with its effectiveness closely tied to training intensity, supervision quality, and patient adherence [17-19]. A Cochrane review confirms that structured PFMT during pregnancy can effectively prevent UI in later pregnancy stages and postpartum [20]. However, the implementation of PFMT faces significant challenges. Research indicates that only 27% of pregnant women are aware of PFMT, while 87.5% perceive UI as a normal pregnancy occurrence [21]. Furthermore, merely 13.1% of affected women seek professional help [22], and a striking 10.7% engage in irregular PFMT during pregnancy [23]. Our previous study [24] demonstrated that unsupervised, low-intensity PFMT failed to improve postpartum UI symptoms, highlighting the need for more effective intervention strategies.

The advent of mobile health (mHealth) technologies has revolutionized chronic disease management and behavioral modification approaches [25]. Mobile apps have demonstrated promise in enhancing UI management effectiveness among women, particularly in improving PFMT adherence and reducing UI symptoms [26-28]. Studies indicate that women with UI view mobile apps favorably as accessible tools that enhance their motivation and sense of empowerment to perform PFMT [29,30]. However, existing research predominantly consists of single-center, small-sample studies focusing on treatment rather than prevention, with limited inclusion of pregnant women [31-33]. Our team previously developed the Urinary Incontinence for Women (UIW) mobile app and evaluated its preliminary effectiveness through a single-center pilot study [34]. While the initial results showed promise, the generalizability of findings was limited by the homogeneous population and specific health care setting. Additionally, the modest intervention effects observed suggested the need for protocol optimization. To address these limitations and generate more robust evidence, we designed this multicenter pragmatic randomized controlled trial to rigorously evaluate the effectiveness of our mobile app–based self-management intervention for maternal UI. The pragmatic design was deliberately chosen to evaluate the intervention under real-world clinical conditions, prioritizing ecological validity and generalizability of findings to routine practice settings. Our goal was to establish evidence-based strategies for early prevention and management of UI during pregnancy that could be readily implemented in everyday clinical practice.

## Methods

### Study Design

We conducted a multicenter, unblinded, parallel-group pragmatic randomized controlled trial comparing an app-based self-management intervention with standard care for UI in pregnant women at a 1:1 allocation ratio. The trial was implemented across 5 hospitals in China. This study adhered to the CONSORT-EHEALTH (Consolidated Standards of Reporting Trials of Electronic and Mobile HEalth Applications and onLine TeleHealth) V1.6.1 guidelines for reporting.

### Study Settings and Participants

The trial was conducted between June and September 2022 at 5 hospitals in China: Dongguan Maternal and Child Health Hospital (center A), Affiliated Hospital of Xiangnan University (center B), Qinghai Kangle Hospital (center C), Foshan Shunde District Third People’s Hospital (center D), and Shenzhen Hospital of Southern Medical University (coordinating center, center E). Participants were recruited from the obstetrics outpatient clinics during routine prenatal visits.

Eligible participants were pregnant women who met the following criteria: (1) aged 18 years or older, (2) singleton pregnancy at 24‐26 weeks of gestation confirmed by ultrasonography, and (3) ownership of and ability to operate a smartphone with web-based access. Women were excluded if they had (1) mental illness or cognitive impairment, (2) medical or surgical conditions that could interfere with study completion, (3) high-risk pregnancy condition contraindicating PFMT (including hypertensive disorders of pregnancy, pregnancy-associated heart disease, preterm labor, habitual abortion, or placenta previa), or (4) history of prepregnancy UI, pelvic organ prolapse, anterior or posterior vaginal wall prolapse, or pelvic surgery.

### Sample Size Determination

Sample size calculations were performed using PASS 2021 software (NCSS) [35]. Based on preliminary data from an exploratory trial [34], which indicated a mean difference of 2.68 and an SD of 4.38 for the primary outcome, the intracluster correlation coefficient was estimated at 0.10 [36]. A 2-sided significance level of 5% and a power of 90% were assumed. Assuming an equal allocation ratio (1:1), the required sample size for each group was determined to be 102 participants. To compensate for an anticipated 20% attrition rate, we increased the sample size to 128 participants per group, yielding a total target enrollment of 256 participants.

### Randomization, Allocation Concealment, and Blinding

This study used simple randomization. Eligible participants were randomly assigned to either the intervention group or the control group using a computer-generated random number sequence. An independent research assistant (DZ), not involved in recruitment or intervention delivery, performed the randomization using a 1:1 allocation ratio. Assignment results were sealed in sequentially numbered, opaque envelopes to ensure allocation concealment. Upon participant enrollment, research assistants opened the envelopes sequentially to reveal group assignments. Given the nature of the intervention, blinding of participants and intervention providers was not feasible.

### Interventions

#### Control Group

Participants in the control group received standard care, including one-on-one health education delivered by trained health care providers. The education covered UI etiology and risk factors, PFMT benefits, and detailed exercise instructions. During the initial session, participants received hands-on PFMT guidance in a supine position. The provider used bimanual palpation (one hand on the perineum and the other on the lower abdomen) to assess the quality of pelvic floor muscle contractions. Specifically, the hand positioned at the perineum directly assessed the characteristic inward and upward movement of the perineal body, which indicates proper recruitment of the levator ani muscle complex. Concurrently, the hand placed on the lower abdomen monitors for unwanted compensatory strategies, including inappropriate abdominal muscle cocontraction, Valsalva maneuvers, or breath-holding patterns that frequently occur during incorrect attempts at pelvic floor activation. This technique provides real-time tactile feedback, enabling immediate correction and ensuring that participants activate the pelvic floor muscles accurately and in isolation, facilitating neuromuscular reeducation and preventing the recruitment of accessory muscle groups that could diminish PFMT effectiveness. The prescribed PFMT protocol consisted of contracting the muscles for 6 seconds, followed by a 6-second relaxation period, repeated 8 times. The goal was to gradually increase each contraction to 15 seconds, with a 5-second relaxation, and participants were encouraged to increase repetitions as tolerated. Daily practice of at least 3 sets was encouraged, with a recommended duration of 12 weeks. Exercises could be performed in any comfortable position.

#### Intervention Group

In addition to standard care, participants in the intervention group received a 12-week self-management program delivered through the “UIW” mobile app (registration number: 2019SR1342273). The comprehensive digital platform includes a user interface and a back-end management system (Multimedia Appendix 1), with four core modules in the user-facing component: (1) Risk assessment module: Using our postpartum incontinence prediction tool [36], this module assesses the risk of postpartum incontinence based on pregnant women’s clinical characteristics. It provides feedback on individual risk levels to help identify high-risk groups. (2) Health knowledge module: This module covers topics such as UI, weight management, pregnancy-related constipation, pelvic floor muscle health, and general pregnancy care. Information is delivered via papers, images, and videos. (3) Pelvic floor muscle training module: This module includes a 2-stage beta version as well as a comprehensive 4-stage PFMT program. Full details of the specific PFMT program are available in our previously published report [34]. (4) Online assessment module: This module uses a validated scale to evaluate UI symptoms, quality of life, and self-efficacy in PFMT.

The back-end system automatically recorded user activity metrics, including login frequency, completed PFMT sessions, and visits to each module. These data enabled researchers to monitor engagement throughout the intervention period. Participants received initial guidance on app installation, registration, and feature navigation. They were encouraged to complete at least 3 PFMT sessions daily using the app for 12 weeks. Additionally, the app sent 3 daily reminders to prompt them to engage in the training.

### Data Collection and Outcome Measurements

Baseline demographic and clinical data were collected at enrollment, including age, education level, height, weight, obstetric history, UI at baseline, and PFMT performing before enrollment (assessed by inquiring whether participants had engaged in PFMT prior to joining the study). Postpartum follow-up at 6‐8 weeks captured delivery-related information (gestational age at birth, mode of delivery, perineal injury, and neonatal birth weight). All outcomes were assessed at 3 time points: baseline (T0), immediately after 12 weeks of intervention (T1), and 6‐8 weeks postpartum (T2), using electronic questionnaires. Participants who delivered or miscarried before completing the 12-week intervention discontinued the program on the day of the event. Data collected up to that point were retained for the intention-to-treat analysis, and missing outcome data were handled according to the predefined strategy.

### Primary Outcomes

The primary outcome was UI symptom severity, measured using the Chinese version of the International Consultation on Incontinence Questionnaire—Urinary Incontinence Short Form [37,38]. This validated scale includes items assessing leak frequency, volume, timing, and the impact on daily life. Total scores range from 0 to 21, with severity categorized as 0‐7 for mild, 8‐13 for moderate, and 14‐21 for severe UI.

### Secondary Outcomes

The impact of UI on quality of life was assessed using the Incontinence Impact Questionnaire-7 [39], which includes 7 items across 4 domains: physical activity, travel, social activity, and mood. The total score ranges from 0 to 21, with higher scores indicating a greater impact on quality of life. The Chinese version of the Incontinence Impact Questionnaire-7 demonstrated good reliability (Cronbach α=0.824) [40].

The Broome Pelvic Muscle Self-Efficacy Scale (BPMSES) was used to evaluate the self-efficacy of the PFMT [41]. The BPMSES comprises 2 dimensions: expected self-efficacy and expected outcomes. It consists of 23 items, scored from 0 to 100, with higher scores indicating greater self-efficacy. The Chinese version of the BPMSES has shown excellent reliability and validity (Cronbach α=0.912) [42].

### Statistical Analysis

All data were analyzed using IBM SPSS Statistics (version 27.0; IBM Corp) and R software (version 4.3.0; R Foundation for Statistical Computing). Continuous variables were presented as means and standard deviations, while categorical variables were reported as frequencies and percentages. The study data were first assessed for normality and then sociodemographic characteristics and baseline outcome variables were compared between the intervention and control groups using 2-tailed independent-samples *t* tests or chi-square tests, as appropriate.

Generalized estimating equation (GEE) modeling is a robust method for analyzing repeated-measures data, allowing for accurate assessment of intervention effects and their interactions with time, even in the presence of missing data [43]. An intention-to-treat analysis was conducted for all participants. The impact of the intervention on both primary and secondary outcomes was evaluated using GEE with an unstructured working correlation matrix. The control group, immediately after 12 weeks of intervention (T1), and center A were used as reference categories. T1 (immediately after 12 weeks of intervention) was selected as the reference time point, instead of baseline (T0), to facilitate the evaluation of whether the effects observed at the end of the intervention were maintained or changed during the postpartum follow-up period (T2). Covariates, including age, history of vaginal delivery, history of abortion, mode of delivery, perineal injury, neonatal birth weight, UI at baseline, and baseline measures of outcomes, were included in the GEE model. A *P *value of <.05 was considered to indicate statistical significance.

Post hoc exploratory subgroup analyses were conducted to examine intervention effects across different baseline outcome levels, without correction for multiple testing. Additionally, sensitivity analyses were conducted using multiple imputation to address potential bias from missing data, particularly for delivery-related variables (mode of delivery, perineal injury, and neonatal birth weight) and outcome measures (UI symptom severity, impact of UI on quality of life, and self-efficacy with PFMT). A supplementary GEE analysis was also performed using baseline (T0) as the reference category.

### Ethical Considerations

The trial was approved by the ethics committee of Shenzhen Hospital, Southern Medical University (NYSZYYEC20210007), which served as the lead ethics review board. All participating centers received approval from their respective ethics committees and formally accepted the lead board’s approval. The study protocol was registered with the Chinese Clinical Trial Registry (ChiCTR2100047533). No deviations from the registered protocol occurred during the study. Participants did not receive any financial or material compensation for their participation in this study. Written informed consent was obtained from all participants prior to enrollment. All data were anonymized and securely stored to ensure the protection of participants’ privacy.

## Results

### Participant Flow

**Figure 1.**
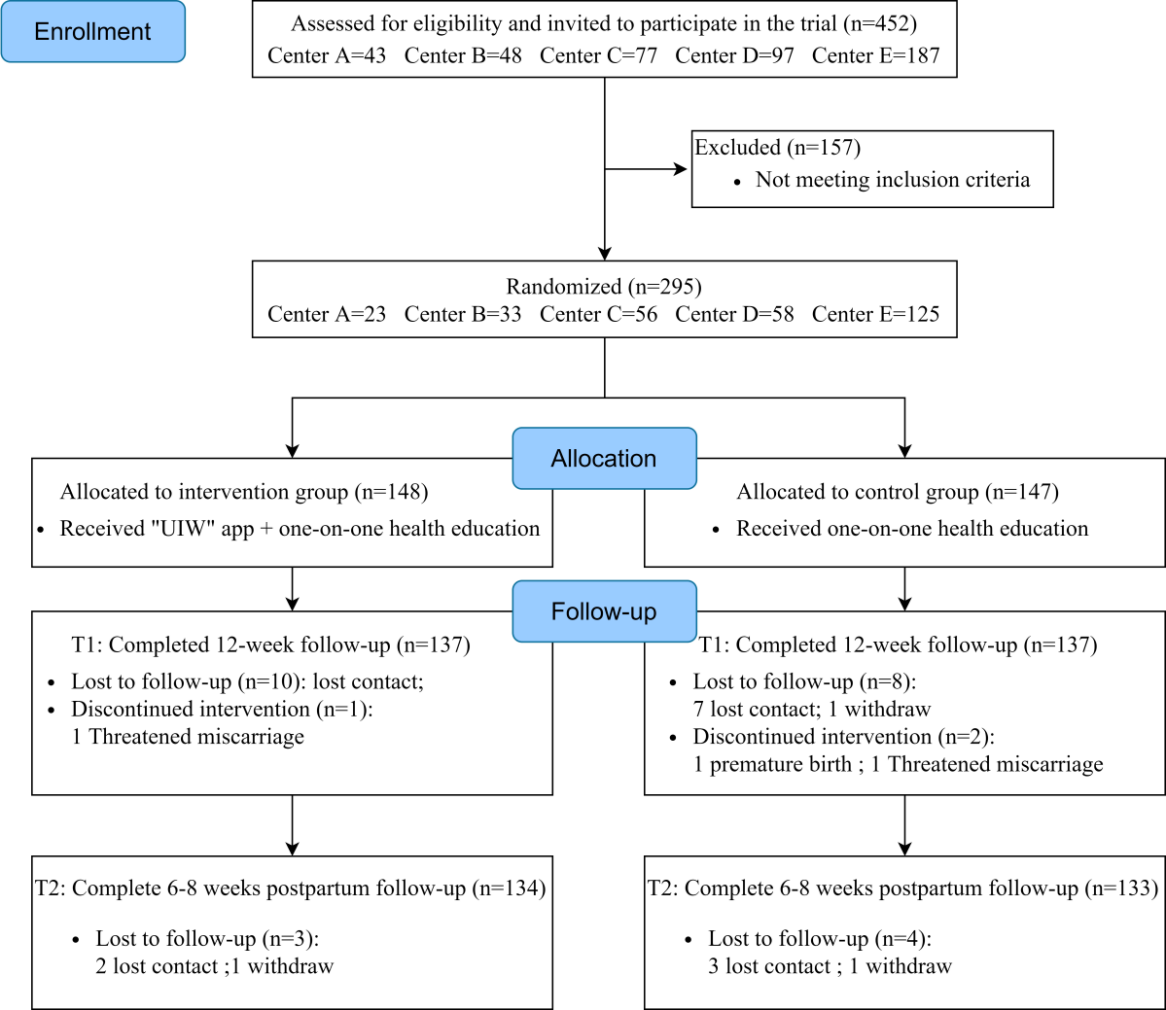
Flowchart of participant recruitment and participation. Center A: Dongguan Maternal and Child Health Hospital; Center B: Affiliated Hospital of Xiangnan University; Center C: Qinghai Kangle Hospital; Center D: Foshan Shunde District Third People’s Hospital; Center E: Shenzhen Hospital of Southern Medical University; PFMT: pelvic floor muscle training; UIW: Urinary Incontinence for Women.

A total of 452 pregnant women were screened for eligibility across 5 hospitals, of whom 295 met the inclusion criteria, provided informed consent, and were randomly assigned to either the intervention group (n=148) or the control group (n=147). Of the 295 participants, 92.9% (274) completed data collection at T1, and 90.5% (267) completed data collection at all time points (Figure 1). A 9% attrition rate was observed in both groups (n=14). Except for lower PFMT self-efficacy scores among participants lost to follow-up (*P*=.02), no significant differences in baseline characteristics were identified between those who completed the study and those who were lost (all *P*>.05) (Table S1 in Multimedia Appendix 2).

### Baseline Characteristics

The mean age of participants was 29.01 years (SD 5.08). Of these, 40.3% (119/295) held a bachelor’s degree or higher, 50.8% (150/295) were primigravida, 46.6% (137/295) had experienced UI prior to enrollment, and only 14.2% (42/295) had practiced PFMT performing before enrollment. At baseline, the intervention group had a higher incidence of vaginal deliveries and perineal injuries than the control group (*P*<.05). Baseline outcome scores showed no statistically significant differences between the groups (*P*>.05). Table 1 shows the baseline characteristics and outcome variables of all participants.

**Table 1.** Baseline characteristics of participants in the intervention and control groups.

Characteristics	Control (n=147)	Intervention (n=148)	Total (n=295)	*t* test (*df*)/Chi-square (*df*)	*P* value
Age (years), mean (SD)	29.05 (5.08)	28.97 (5.10)	29.01 (5.08)	0.13 (293)^a^	.90
Education level, n (%)					
Senior high school or below	50 (34.0)	43 (29.1)	93 (31.5)	0.84 (2)^b^	.66
Junior college	40 (27.2)	43 (29.1)	83 (28.1)		
Bachelor’s degree or above	57 (38.8)	62 (41.9)	119 (40.3)		
BMI at baseline, mean (SD)	23.86 (3.47)	23.66 (3.52)	23.76 (3.49)	0.51 (293)^a^	.61
Primigravida, n (%)					
No	74 (50.3)	71 (48.0)	145 (49.2)	0.17 (1)^b^	.68
Yes	73 (49.7)	77 (52.0)	150 (50.8)		
History of vaginal delivery, n (%)					
No	103 (70.1)	111 (75.0)	214 (72.5)	0.90 (1)^b^	.34
Yes	44 (29.9)	37 (25.0)	81 (27.5)		
History of abortion, n (%)					
No	101 (68.7)	96 (64.9)	197 (66.8)	0.49 (1)^b^	.48
Yes	46 (31.3)	52 (35.1)	98 (33.2)		
History of cesarean section, n (%)					
No	124 (84.4)	133 (89.9)	257 (87.1)	2.00 (1)^b^	.16
Yes	23 (15.6)	15 (10.1)	38 (12.9)		
UI^c^ at baseline^d^, n (%)					
No	83 (56.5)	75 (50.7)	158 (53.6)	0.99 (1)^b^	.32
Yes	64 (43.5)	73 (49.3)	137 (46.4)		
PFMT^e^ performing before enrollment, n (%)					
No	127 (86.4)	126 (85.1)	253 (85.8)	0.10 (1)^b^	.76
Yes	20 (13.6)	22 (14.9)	42 (14.2)		
Mode of delivery^f^, n (%)					
Vaginal delivery	88 (59.9)	105 (70.9)	193 (65.4)	4.95 (1)^b^	.03
Cesarean section	45 (30.6)	29 (19.6)	74 (25.1)		
Perineal injury^f^, n (%)					
No	89 (60.5)	73 (49.3)	162 (54.9)	4.33 (1)^b^	.04
Yes	44 (29.9)	61 (41.2)	105 (35.6)		
Gestational age at birth ^f^, mean (SD)	39.33 (1.19)	39.45 (1.23)	39.39 (1.21)	−0.79 (265)^a^	.43
Neonatal birth weight^f^, mean (SD)	3.29 (0.45)	3.27 (0.38)	3.28 (0.42)	0.39 (265)^a^	.69
UI symptom severity^g^ (ICIQ−UI−SF^h^ score), mean (SD)	3.03 (4.11)	3.24 (3.75)	3.14 (3.93)	−0.46 (293)^a^	.65
Impact of UI on quality of life^g^ (IIQ-7^i^ score), mean (SD)	1.21 (3.31)	1.44 (3.35)	1.33 (3.33)	−0.59 (293)^a^	.56
Self−efficacy with PFMT^j^ (BPMSES^k^ score), mean (SD)	69.06 (23.88)	68.93 (23.22)	68.99 (23.51)	0.45 (293)^a^	.96

a Independent 2-sample *t* test*.*

b Chi-square test.

c UI: urinary incontinence.

d An International Consultation on Incontinence Questionnaire—Urinary Incontinence Short Form (ICIQ-UI-SF) score of 0 indicates no urinary incontinence at enrollment, whereas a nonzero score indicates urinary incontinence *at* enrollment.

e PFMT: pelvic floor muscle training.

f 28 missing.

g A higher score indicates a worse outcome.

h ICIQ-UI-SF: International Consultation on Incontinence Questionnaire—Urinary Incontinence Short Form.

i IIQ-7: Incontinence Impact Questionnaire-7.

j A higher score indicates a better outcome;

k BPMSES: Broome Pelvic Muscle Self-Efficacy Scale.

### PFMT Session Completion Records

Of the 148 participants assigned to the intervention group, 92.6% (137) completed the full 12-week program. Throughout the intervention period, these participants collectively completed 14,936 PFMT sessions (mean 126.77, SD 43.39; range 41‐233 sessions), with a cumulative training duration of 29,062 minutes (mean 245.12, SD 84.34; range 80.80‐466.10 minutes).

### Outcomes

#### Primary Outcome

**Figure 2.**
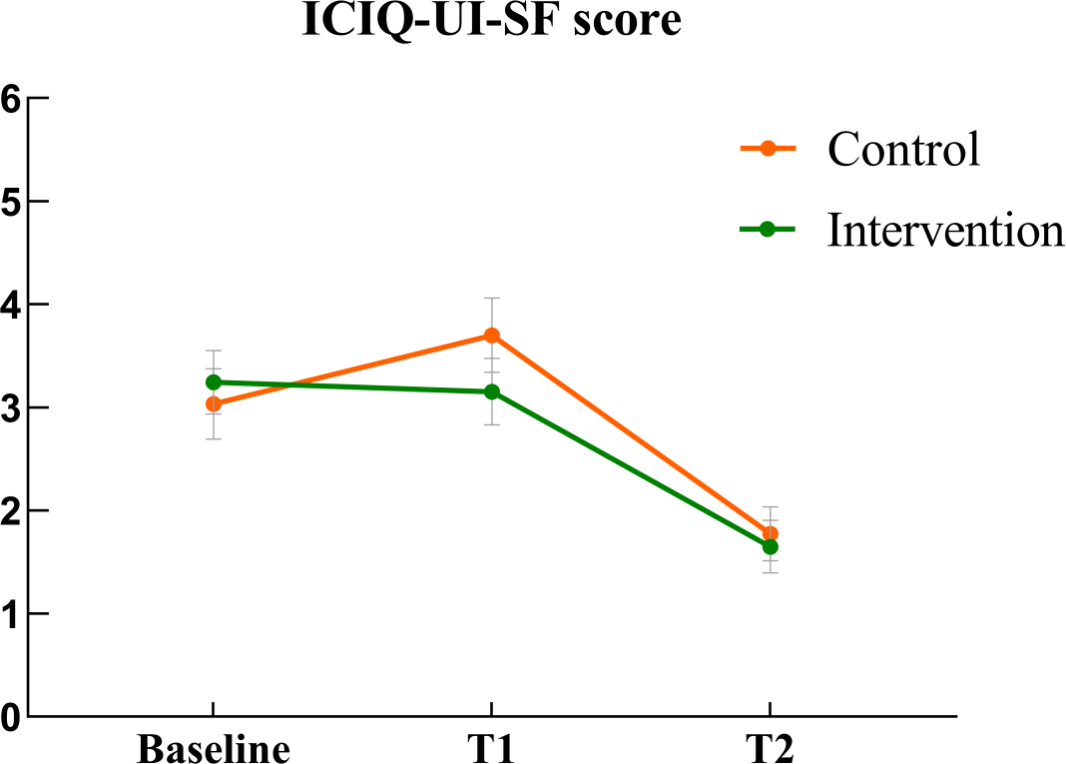
Mean and standard error of urinary incontinence symptom severity in both groups across the 3 time points. ICIQ-UI-SF: International Consultation on Incontinence Questionnaire—Urinary Incontinence Short Form; T1: immediately after 12 weeks of intervention; T2: 6‐8 weeks postpartum.

UI symptom severity in the control group initially increased before later decreasing, while the intervention group showed a consistent decline over time (Figure 2). The GEE analysis indicated a significant difference between the intervention and control groups (β=−.97; *P*=.03). A significant improvement in UI symptom severity was observed at 6‐8 weeks postpartum (β=−1.93; *P*<.001), with immediately after 12 weeks of intervention (T1) as the reference. Compared with center A, all centers except center B demonstrated statistically significant differences (*P*<.05). Table 2 shows the results of the GEE analysis of the primary outcome.

**Table 2.** Generalized estimating equation models of the comparison of primary outcome between the intervention and control groups.

Outcome variables^a^	SE	β (95% CI)	*P* value
UI^b^ symptom severity^c^			
Group effect			
Control (reference)			
Intervention	0.45	−.97 (−1.85 to −0.08)	.03
Time effect			
T1^d^ (reference)			
T2^e^	0.39	−1.93 (−2.70 to −1.17)	<.001
Center effect			
Center A (reference)			
Center B	0.70	−.97 (−2.34 to −0.40)	.17
Center C	0.59	−1.32 (−2.49 to −0.16)	.03
Center D	0.56	−1.46 (−2.56 to −0.37)	.009
Center E	0.47	−1.43 (−2.35 to −0.51)	.002
Group × Time effect			
Control (reference)			
Intervention × T2	0.52	.55 (−0.47 to 1.58)	.29

a Age, history of vaginal delivery, history of abortion, mode of delivery, perineal injury, neonatal birth weight*,* urinary incontinence at baseline, and baseline score as adjusted covariates.

b UI: urinary incontinence.

c A higher score indicates a worse outcome.

d Immediately after 12 weeks of intervention.

e Six to eight weeks postpartum.

#### Secondary Outcomes

**Figure 3.**
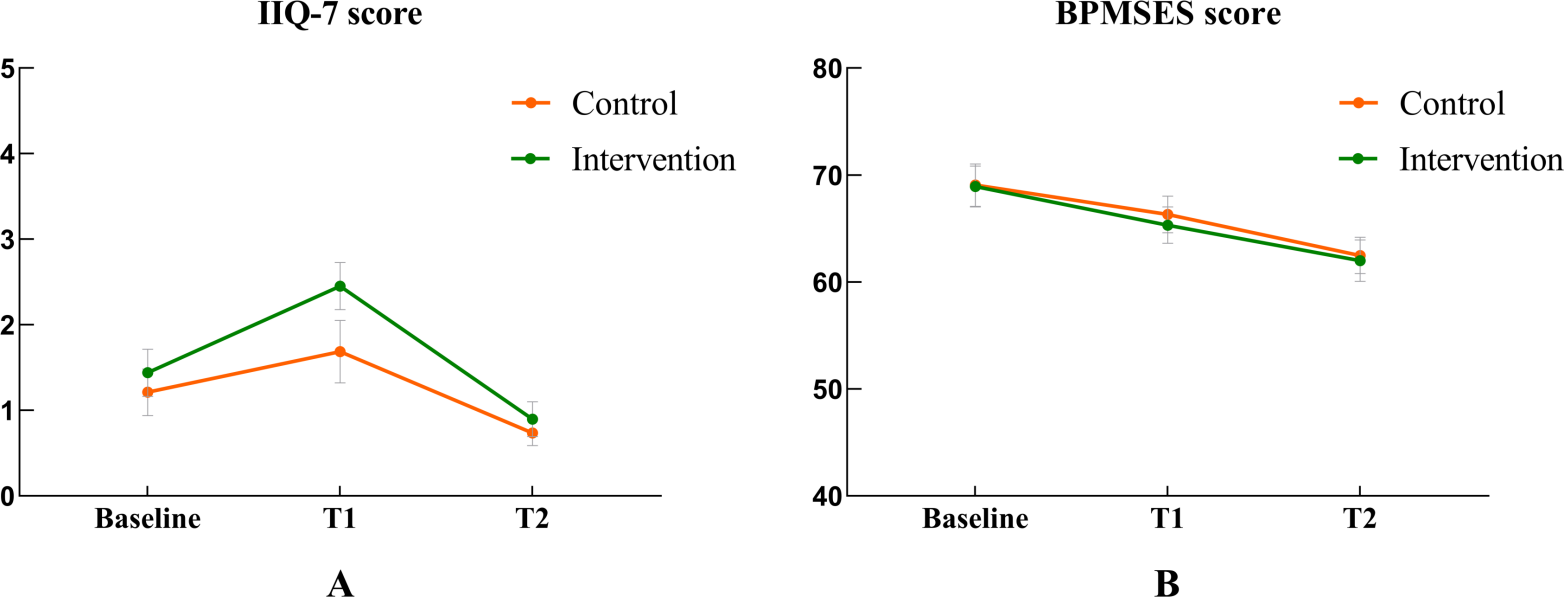
Mean and standard error of (A) quality of life and (B) pelvic floor muscle training self-efficacy in both groups across the study period. BPMSES: Broome Pelvic Muscle Self-Efficacy Scale; IIQ-7: Incontinence Impact Questionnaire-7; T1: immediately after 12 weeks of intervention; T2: 6‐8 weeks postpartum.

Quality-of-life scores initially increased in both the control and intervention groups but subsequently declined over time, while self-efficacy with PFMT demonstrated a steady decrease across both groups throughout the follow-up period (Figure 3). A significant difference in quality of life was observed between the intervention and control groups (β=−1.05; *P*=.048). Quality of life improved significantly at 6‐8 weeks postpartum (β=−1.72; *P*<.001), using the immediate post–12-week intervention period (T1) as a reference. Additionally, a significant difference was found between center C and center E (*P*<.05). Although no significant between-group difference was observed for self-efficacy with PFMT (β=1.49; *P*=.51), self-efficacy scores declined significantly at 6‐8 weeks postpartum (β=−4.66; *P*=.008), with a notable difference between center D and center E (*P*<.05). Table 3 shows the results of the GEE analysis of the secondary outcomes.

**Table 3.** Generalized estimating equation models of the comparison of secondary outcomes between the intervention and control groups.

Outcome variables^a^	SE	β (95% CI)	*P* value
Impact of UI^b^ on quality of life^c^			
Group effect			
Control (reference)			
Intervention	0.46	−1.05 (−1.95 to −0.15)	.048
Time effect			
T1^d^ (reference)			
T2^e^	0.38	−1.72 (−2.46 to −0.98)	<.001
Center effect			
Center A (reference)			
Center B	0.72	−1.31 (−2.73 to 0.10)	.07
Center C	0.63	−1.88 (−3.12 to −0.64)	.04
Center D	0.66	−1.66 (−2.96 to −0.36)	.05
Center E	0.59	−2.15 (−3.30 to −0.99)	.04
Group × Time effect			
Control (reference)			
Intervention × T2	0.49	.97 (0.01 to 1.93)	.048
Self-efficacy with PFMT^f^^,g^			
Group effect			
Control (reference)			
Intervention	2.25	1.49 (−2.93 to 5.90)	.51
Time effect			
T1^d^ (reference)			
T2^e^	1.76	−4.66 (−8.11 to −1.22)	.008
Center effect			
Center A (reference)			
Center B	5.13	4.00 (−6.05 to 14.05)	.44
Center C	3.36	−3.14 (−9.73 to 3.45)	.35
Center D	3.09	18.52 (12.46 to 24.58)	<.001
Center E	2.97	12.12 (6.31 to 17.94)	<.001
Group × Time effect			
Control (reference)			
Intervention × T2	1.56	2.42 (−3.19 to 6.31)	.52

a Age, history of vaginal delivery, history of abortion, mode of delivery, perineal injury, neonatal birth weight*,* urinary incontinence at baseline, and baseline score (T0) as adjusted covariates.

b UI: urinary incontinence.

c A higher score indicates a worse outcome.

d Immediately after 12 weeks of intervention*.*

e Six to eight weeks postpartum.

f PFMT: pelvic floor muscle training.

g A higher score indicates a better outcome.

### Subgroup Analyses

**Figure 4.**
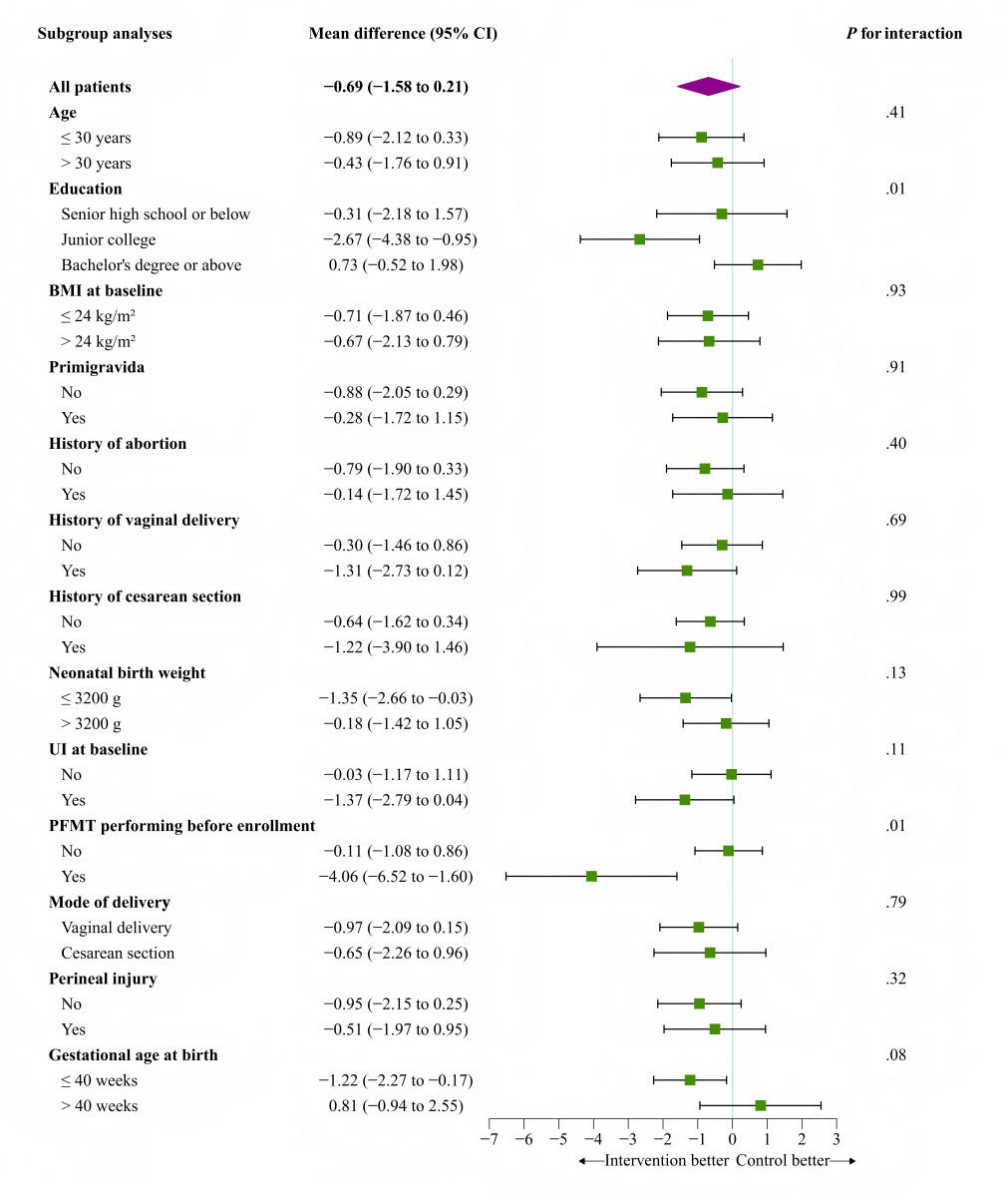
Subgroup analysis of primary outcome of urinary incontinence symptom severity at immediate 12 weeks of intervention. Center and baseline score as adjusted covariates. PFMT: pelvic floor muscle training; UI: urinary incontinence.

An exploratory subgroup analysis was conducted on the severity of UI symptoms as the primary outcome at 12 weeks postintervention and 6‐8 weeks postpartum (Figures4  5). The analysis revealed statistically significant interactions between the intervention effect and education level (*P*=.01) as well as PFMT performing before enrollment (*P*=.01) at 12 weeks postintervention. Furthermore, at 6‐8 weeks postpartum, significant interactions were identified between the intervention effect and education level (*P*=.01), history of vaginal delivery (*P*=.048), and UI symptoms at baseline (*P*=.02).

**Figure 5.**
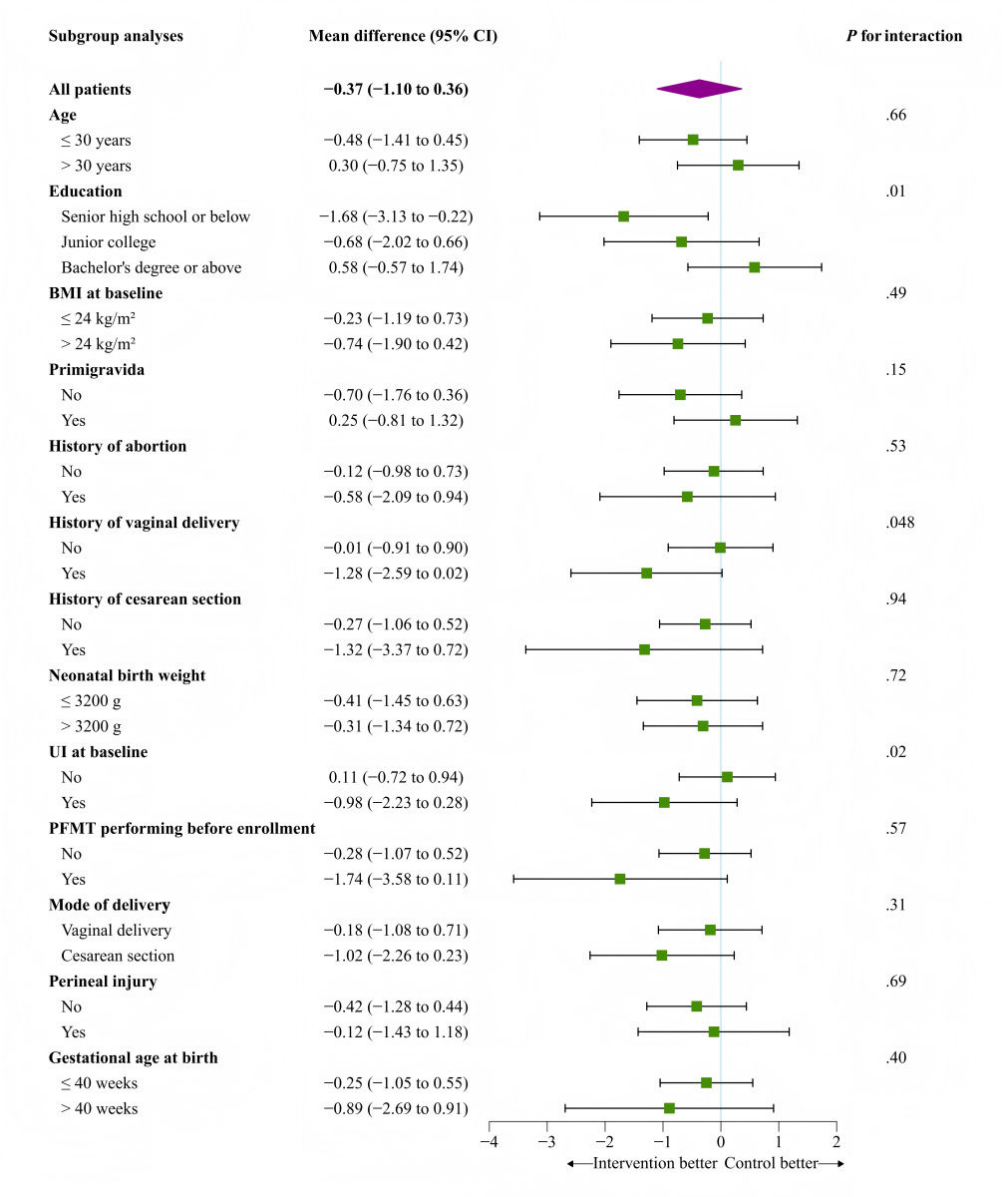
Subgroup analysis of primary outcome of urinary incontinence symptom severity at 6‐8 weeks postpartum. Center and baseline score as adjusted covariates. PFMT: pelvic floor muscle training; UI: Urinary incontinence.

### Sensitivity Analysis

The sensitivity analysis, conducted using the multiple imputation method, showed results that were generally consistent with the primary analysis (Table S2 in Multimedia Appendix 2). Significant improvements were observed in both the group effect (*P*=.04) and the time effect (*P*<.001) in relation to UI symptom severity. In addition, a supplementary GEE analysis using baseline (T0) as the reference point also demonstrated consistent findings. The intervention group showed a significant improvement in UI symptom severity compared with the control group (*P*=.04), with a further significant decline observed at 6‐8 weeks postpartum compared with baseline (*P*<.001) (Table S3 in Multimedia Appendix 2).

## Discussion

### Principal Findings

To our knowledge, this multicenter randomized controlled trial represents the first comprehensive evaluation of an mHealth intervention for UI self-management during pregnancy in China. By recruiting participants from diverse regional areas, we minimized potential biases inherent in single-center studies, allowing for the collection of more representative data and enhancing the scope, authenticity, and reliability of our findings. Notably, the follow-up completion rate was 90.5%, which is highly favorable for a multicenter trial. The high follow-up rate strengthens the robustness of our conclusions, although the lower baseline self-efficacy among those lost to follow-up suggests challenges in engaging less confident users.

Significant improvement in UI symptom severity was observed in the intervention group. These findings align with those of a systematic review [44], which, although limited to women with UI, similarly demonstrated the effectiveness of mobile apps in reducing UI symptom severity. Our findings contrast with a study of primiparous women using a different mobile app [45], which found no significant difference between groups. This inconsistency may be attributed to variations in app functionality: our “UIW” app incorporates video explanations and animated instructions, potentially facilitating more intuitive and accurate PFMT performance, particularly for those unfamiliar with the exercises. The previous study’s focus on primiparous women, who generally experience milder UI [45], and their longer follow-up period (6 months vs our 12 weeks and postpartum assessment) might also explain the differing results. It is important to note that our study included women both with and with no preexisting UI (as indicated in the baseline characteristics), broadening the potential applicability of our findings.

The intervention group also experienced a significant improvement in UI-related quality of life, consistent with other studies demonstrating the positive impact of mHealth programs on quality of life in individuals with UI [26,45]. Unlike prior research that primarily targeted middle-aged and older adult women with existing UI, our study included pregnant women irrespective of their UI status. By incorporating PFMT as a self-management strategy, the intervention helped women better manage UI symptoms, thereby enhancing quality of life. While pregnancy-related physiological changes and postpartum spontaneous recovery are known to affect UI patterns [10,46], the significantly greater improvements observed in the intervention group than in the control group (who would have experienced the same natural recovery) suggest that our intervention provided benefits beyond natural processes. Importantly, our subgroup analyses revealed that the mobile app–based PFMT appears to be more beneficial for women who already experienced UI symptoms during pregnancy, with little evidence supporting its preventive effect in asymptomatic women. This suggests that clinical resources might be more efficiently used by prioritizing such interventions for symptomatic pregnant women.

An unexpected finding of our study was the significant decline in PFMT self-efficacy over time across both groups. While evidence suggests that higher self-efficacy typically predicts better PFMT adherence [47,48], our findings parallel those of a previous study that reported no significant improvement in self-efficacy among pregnant women with UI following a mobile app intervention at the 2-month follow-up, compared with the 1-month postintervention [49]. This outcome contrasts with our earlier single-center trial [34], where participants maintained higher self-efficacy throughout the follow-up period. Multiple factors likely contributed to this finding: (1) The 12-week intervention duration, which exceeds our previous 8-week program [34], may have induced behavioral fatigue with a gradual decline in perceived competence; and (2) low adherence to the recommended training protocol, as evidenced by our backend usage data, which showed declining engagement with the PFMT module despite daily reminders and clear instructions to complete at least 3 PFMT sessions daily. Recent research has revealed that adherence is closely associated with PFMT self-efficacy [50], suggesting a bidirectional relationship where reduced practice leads to decreased confidence in one’s ability to perform PFMT correctly. Importantly, the paradox of UI symptom relief and quality-of-life gains alongside falling self-efficacy suggests that even suboptimal practice can yield clinical benefits, although perhaps not to its full potential. Future digital interventions for UI should incorporate real-time adherence monitoring and targeted motivators to sustain both exercise performance and users’ confidence in their ability to perform PFMT correctly.

Subgroup analyses revealed important patterns in intervention effectiveness across participant characteristics, highlighting the need for targeted approaches in UI management. In particular, by comparing women with and with no antenatal UI, we were able to assess both treatment and preventive effects: the intervention produced substantial symptom improvement in the antenatal‐UI subgroup, whereas changes in asymptomatic women were more modest, suggesting stronger therapeutic than preventive efficacy. Our study also identified dynamic moderating effects of educational level on intervention outcomes. At 12 weeks postintervention, participants with junior college education demonstrated significant improvement, whereas those with senior high school or lower education exhibited more pronounced benefits at 6‐8 weeks postpartum. This finding contrasts with a previous study [51], which suggested that a higher educational level predicted better outcomes in app-based UI management. This difference may reflect variations in eHealth literacy, compliance, and adaptability to app design among people with different educational levels, thus emphasizing the necessity of formulating stratified UI management strategies for different educational-level groups. Prior experience with PFMT also moderated the intervention’s effect at 12 weeks postintervention. Women with prior PFMT experience demonstrated greater improvement in UI symptoms. Existing research suggests that preexisting knowledge and familiarity with pelvic floor exercises may enhance the effectiveness of app-based interventions [52]. This highlights the importance of integrating PFMT education into routine prenatal care. The differential effects observed at 6‐8 weeks postpartum were particularly informative. Although vaginal delivery and antenatal UI symptoms are established risk factors for postpartum UI [13,14], our study found that women with these characteristics demonstrated greater improvement from the app-based intervention. This seemingly paradoxical finding may be explained by several factors. Women with a vaginal delivery history or preexisting UI symptoms may have heightened awareness of pelvic floor dysfunction, leading to stronger motivation and better intervention adherence. Additionally, those with baseline UI symptoms might be more attuned to symptom changes, while women with vaginal delivery may show increased responsiveness to rehabilitation exercises due to childbirth-related muscle changes. Previous research has suggested that symptom severity can positively influence treatment engagement and outcomes [28,53]. These findings suggest that targeted digital interventions can be effective even in traditionally high-risk populations. However, given the exploratory nature of the subgroup analyses and the multiple comparisons performed, no adjustment for multiplicity was applied. As a result, the risk of type I error may be increased, and these findings should be interpreted with caution as hypothesis-generating rather than confirmatory.

Intercenter variability was observed across our 5 study sites. Significant differences between center A (reference) and centers C, D, and E were noted for UI symptom severity; between center A and centers C and E for quality of life; and between center A and centers D and E for PFMT self-efficacy. These variations likely reflect differences in incontinence service conditions, health care resources, and models of care across centers. Future research should investigate the specific factors contributing to these differences and explore strategies for optimizing resources to enhance UI management and improve overall intervention effectiveness.

### Limitations

This study has several limitations. First, data collection relied on participants’ self-reports, which may be affected by recall bias or social desirability, potentially impacting data accuracy. Second, the nature of the intervention precluded participant blinding, which may have introduced expectancy bias. Participants in the intervention group may have anticipated benefits from the application and therefore reported more favorable subjective outcomes, potentially influencing both their behavioral engagement and outcome reporting. Future studies should consider incorporating objective outcome measures to enhance the reliability of the findings. Third, as a pragmatic behavioral intervention, we monitored PFMT session completion only in the intervention group via the app’s back-end system, revealing natural variation in usage patterns. However, we could not directly track adherence in the control group, limiting our ability to compare “intervention dose” across arms. This variability reflects real-world implementation challenges—particularly among pregnant women juggling multiple priorities—and should inform future adaptive designs that integrate objective adherence measures and tailored engagement strategies. Fourth, although the PFMT module provides real-time guidance through column-based graphics, the app lacks motivational features such as interactive feedback, gamification, and peer interaction. This limitation may reduce long-term engagement and adherence. Future versions could consider incorporating these elements to enhance user motivation and intervention effectiveness. Fifth, the multicenter design, while enhancing generalizability, revealed variations in UI service delivery, health care resources, and care models across participating centers that may have impacted intervention effectiveness. Understanding these intercenter differences and their influence on outcomes warrants further investigation. Finally, the relatively short follow-up period limited our ability to assess the intervention’s long-term effects. Future studies should incorporate extended follow-up periods (such as 3 months, 6 months, 1 year, or longer postpartum) to evaluate the sustainability of intervention effects and their long-term impact on UI management. Additionally, the use of mixed methods designs in prospective longitudinal studies is recommended to explore user experiences and to develop more effective adherence support strategies for maintaining long-term engagement and assessing intervention efficacy over time.

### Conclusions

This study provides evidence that the “UIW” mHealth app is effective in reducing UI symptom severity and improving quality of life among pregnant women. However, the lack of improvement in PFMT self-efficacy highlights the need for further research into strategies for enhancing user engagement and long-term adherence. Future studies should incorporate longer follow-up periods and explore the impact of intercenter variability on intervention effectiveness.

## Supplementary material

10.2196/72883 Multimedia Appendix 1 Screenshot of Urinary Incontinence for Women app.

10.2196/72883 Multimedia Appendix 2 Baseline comparison (completers vs lost to follow-up) and sensitivity analyses for the primary outcome.

10.2196/72883 Checklist 1 CONSORT-EHEALTH (Consolidated Standards of Reporting Trials of Electronic and Mobile E-Health Applications and Online Telehealth) V1.6.1 checklist.

## Abbreviations

BPMSES: Broome Pelvic Muscle Self-Efficacy Scale
CONSORT-EHEALTH: Consolidated Standards of Reporting Trials of Electronic and Mobile Health Applications and Online Telehealth
GEE: generalized estimating equation
mHealth: mobile health
PFMT: pelvic floor muscle training
UI: urinary incontinence
UIW: Urinary Incontinence for Women
