# Supplementary material for: Mobile App–Based Self-Management of Urinary Incontinence in Pregnant Women: Multicenter Pragmatic Randomized Controlled Trial
Source: J Med Internet Res. 2025 Aug 7;27:e72883. doi: 10.2196/72883 (PMC12331131; doi:10.2196/72883)
Supplement: Multimedia Appendix 1 [file jmir-v27-e72883-s001.docx]

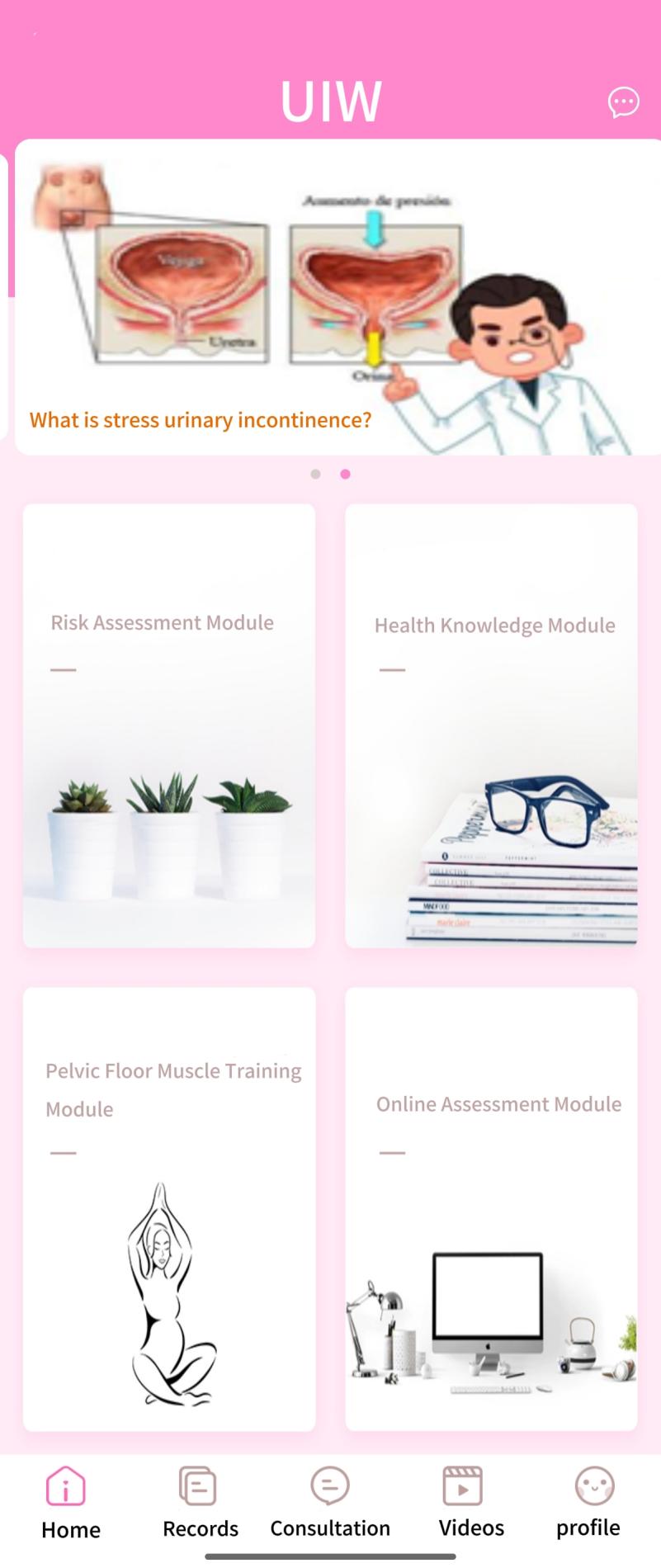


1. UIW App: The user interface encompasses four primary modules. The screenshots have been translated into English.

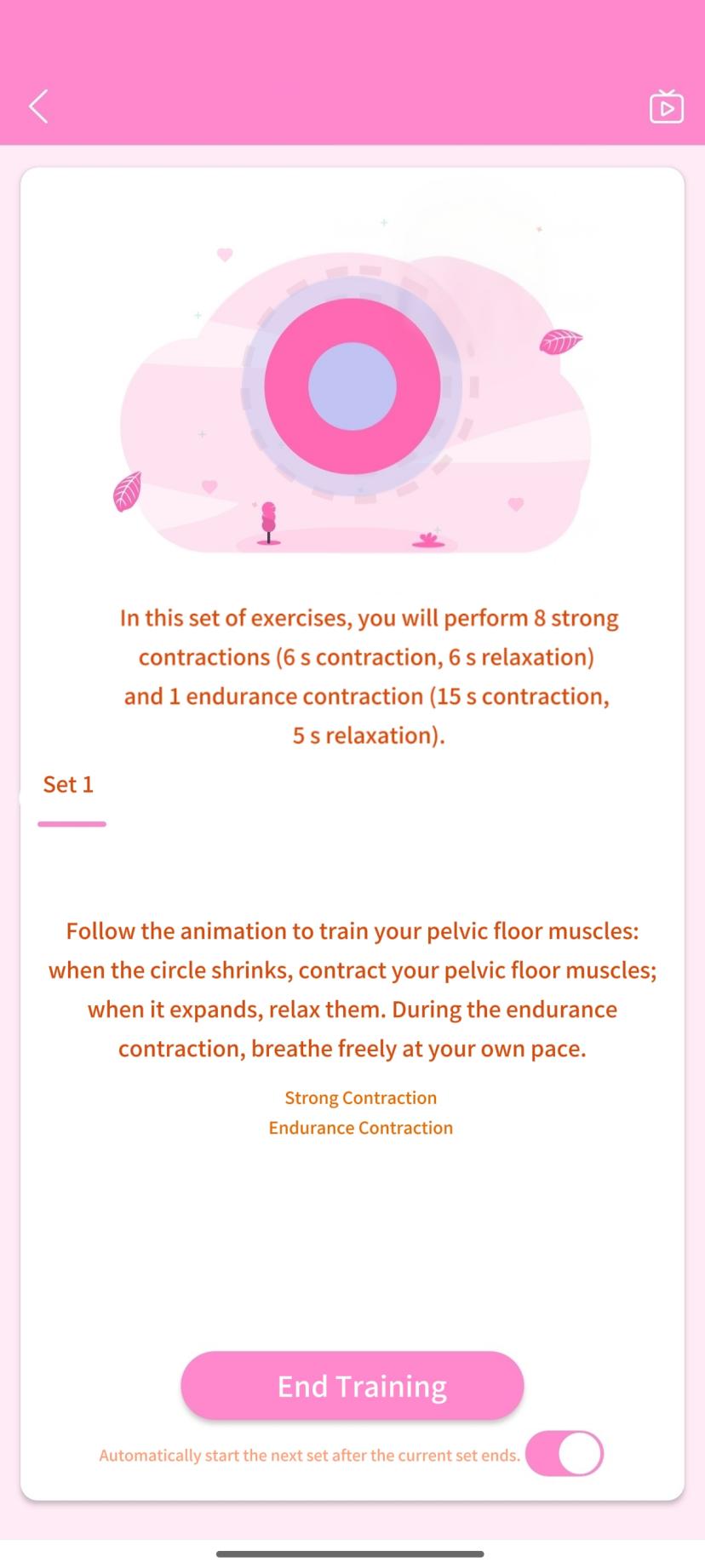

2. Pelvic Floor Muscle Training Module: The app shows real-time, dynamic guidance in columnar graphics, presenting the duration and intensity of pelvic floor muscle contractions with concomitant relaxation. The screenshots have been translated into English.


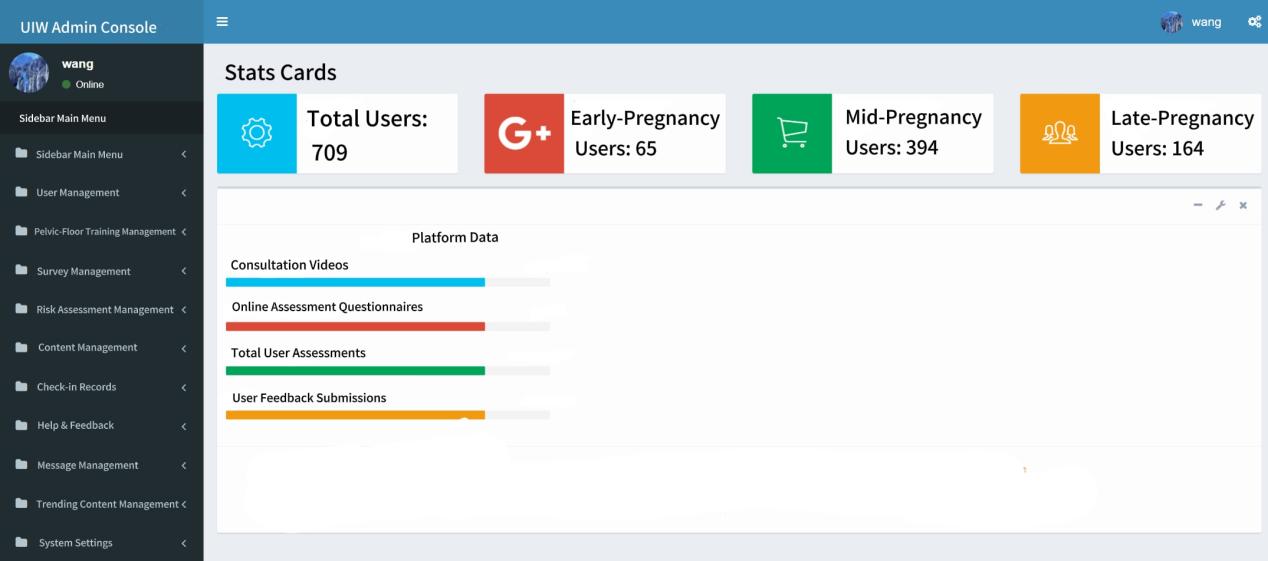


1. UIW Admin Console: This researcher-facing dashboard provides access to user statistics and platform usage data. The screenshots have been translated into English.
