# Supplementary material for: Mobile App–Based Self-Management of Urinary Incontinence in Pregnant Women: Multicenter Pragmatic Randomized Controlled Trial
Source: J Med Internet Res. 2025 Aug 7;27:e72883. doi: 10.2196/72883 (PMC12331131; doi:10.2196/72883)
Supplement: Multimedia Appendix 2 [file jmir-v27-e72883-s002.docx]

**Table S1.** Comparison of baseline characteristics between the participants who completed the study and those who were lost to follow-up.

| Characteristics | Completed (n=267) | lost to follow-up  (n=28) | Total  (n=295) | *t* test (df)/chi-square (df) | *P* value | |
| --- | --- | --- | --- | --- | --- | --- |
| Age (years), mean (SD) | 29.01 (5.19) | 29.00 (3.95) | 29.01 (5.08) | 0.01 (293)^a^ | .99 | |
| **Education level, n(%)** | | | | | | |
| Senior high school or below | 89 (33.3) | 4 (14.3) | 93 (31.5) | 4.44 (2)^b^ | .11 | |
| Junior college | 74 (27.7) | 9 (32.1) | 83 (28.1) |  |  |  |
| Bachelor's degree or above | 104 (39.0) | 15 (53.6) | 119 (40.3) |  |  |  |
| BMI at enrollment, mean (SD) | 23.79 (3.50) | 23.51 (3.40) | 23.76 (3.49) | 0.40 (293)^a^ | .69 | |
| **Primigravida, *n*(%)** | | | | | | |
| No | 132 (49.4) | 13 (46.4) | 145 (49.2) | 0.09 (1)^b^ | .76 | |
| Yes | 135 (50.6) | 15 (53.6) | 150 (50.8) |  |  |  |
| **History of abortion, *n*(%)** | | | | | | |
| No | 192 (71.9) | 22 (78.6） | 214 (72.5) | 0.57 (1)^b^ | .45 | |
| Yes | 75 (28.1) | 6 (21.4） | 81 (27.5) |  |  |  |
| **History of vaginal delivery, *n*(%)** | | | | | | |
| No | 178 (66.7) | 19 (67.9) | 197 (66.8) | 0.02 (1)^b^ | .90 | |
| Yes | 89 (33.3) | 9 (32.1) | 98 (33.2) |  |  |  |
| **History of cesarean section , *n*(%)** | | | | | | |
| No | 231 (86.5) | 26 (92.9) | 257 (87.1) | 0.91 (1)^b^ | .34 | |
| Yes | 36 (13.5) | 2 (7.1) | 38 (12.9) |  |  |  |
| **UI^c^ at baseline^d^, *n*(%)** | | | | | | |
| No | 146 (54.7) | 12 (42.9) | 158 (53.6) | 1.43 (1)^b^ | .23 | |
| Yes | 121 (45.3) | 16 (57.1) | 137 (46.4) |  |  |  |
| **PFMT^e^ performing before enrollment, *n*(%)** | | | | | | |
| No | 228 (85.4) | 25 (89.3) | 253 (85.8) | 0.31 (1)^b^ | .58 | |
| Yes | 39 (14.6) | 3 (10.7) | 42 (14.2) |  |  |  |
| **Mode of delivery^f^, *n*(%)** | | | | | | |
| Vaginal delivery | 193 (72.3) | N/A^g^ | 193 (65.4) | N/A^g^ | N/A^g^ | |
| Cesarean section | 74 (27.7) | N/A^g^ | 74 (25.1) |  |  |  |
| **Perineal injury^f^, *n*(%)** | | | | | | |
| No | 162 (60.7) | N/A^g^ | 162 (54.9) | N/A^g^ | N/A^g^ | |
| Yes | 105 (39.3) | N/A^g^ | 105 (35.6) |  |  |  |
| Gestational age at birth ^f^, mean (SD) | 39.39 (1.21) | N/A^g^ | 39.39 (1.21) | N/A^g^ | N/A^g^ | |
| Neonatal birth weight^f^, mean (SD) | 3.28 (0.42) | N/A^g^ | 3.28 (0.42) | N/A^g^ | N/A^g^ | |
| UI symptom severity^h^ (ICIQ-UI-SF score^i^), mean (SD) | 3.11 (3.97) | 3.39 (3.56) | 3.14 (3.93) | -0.36 (293)^a^ | .72 | |
| \| Impact of UI on quality of life^h^ (IIQ-7 score^j^), mean (SD) \| \| --- \| | 2.05 (3.80) | 2.71 (3.99) | 1.33 (3.33) | -0.45 (293)^a^ | .65 |  |
| Self-efficacy with PFMT^k^ (BPMSES score^l^), mean (SD) | 69.99 (23.40) | 59.43 (22.75) | 68.99 (23.51) | 2.28 (293)^a^ | **.02** |  |

*Note*. ^a^Independent Two-Sample t-test; ^b^Chi-square test; ^c^UI: urinary incontinence; ^d^An International Consultation on Incontinence Questionnaire-Urinary Incontinence Short Form (ICIQ-UI-SF) score of 0 indicates no urinary incontinence at enrollment, whereas a nonzero score indicates urinary incontinence at enrollment; ^e^PFMT: pelvic floor muscle training; ^f^28 missing; ^g^N/A:At 6-8 weeks postpartum, data was missing due to participant loss to follow-up; ^h^A higher score indicates a worse outcome; ^i^ICIQ-UI-SF: International Consultation on Incontinence Questionnaire-Urinary Incontinence Short Form; ^j^IIQ-7: Incontinence Impact Questionnaire-7; ^k^A higher score indicates a better outcome; ^l^BPMSES: Broome Pelvic Muscle Self-Efficacy Scale;

**Table S2.** Generalized estimating equation models for the primary outcome using multiple imputation.

| Outcome variables^a^ |  | *SE* | *β* (95% CI) | *P* value |
| --- | --- | --- | --- | --- |
| **UI^b^ symptom severity^c^** | | | | |
| Group effect | Control (Reference) | | | |
|  | Intervention | 0.42 | -0.85 (-1.68, -0.02) | **.04** |
| Time effect | T1^d^ (Reference) | | | |
|  | T2^e^ | 0.36 | -1.89 (-2.59, -1.19) | **<.001** |
| Center effect | Center A (Reference) | | | |
|  | Center B | 0.64 | -0.94 (-2.20, 0.32) | .15 |
|  | Center C | 0.56 | -1.24 (-2.35, -0.14) | **.03** |
|  | Center D | 0.52 | -1.33 (-2.35, -0.30) | **.01** |
|  | Center E | 0.43 | -1.28 (-2.14, -0.42) | **.003** |
| Group*Time effect | Control (Reference) | | | |
|  | Intervention*T2 | 0.48 | 0.46 (-0.48, 1.41) | .34 |

Note. ^a^A age, history of vaginal delivery, history of abortion, mode of delivery, perineal injury, neonatal birth weight, urinary incontinence at baseline and baseline score as adjusted covariates; ^b^UI：Urinary Incontinence; ^c^A Higher score indicates a worse outcome; ^d^Immediately after 12 weeks of intervention; ^e^Six to eight weeks postpartum.

**Table S3.** Generalized estimating equation models for the primary outcome with baseline as the reference time point.

| Outcome variables^a^ |  | *SE* | *β* (95% CI) | *P* value |
| --- | --- | --- | --- | --- |
| UI^b^ symptom severity^c^ | | | | |
| Group effect | Control (Reference) | | | |
|  | Intervention | 0.18 | -0.37 (-0.73, -0.01) | **.04** |
| Time effect | T0^d^ (Reference) | | | |
|  | T1^e^ | 0.27 | 0.26 (-0.27, 0.78) | .34 |
|  | T2^f^ | 0.27 | -1.40 (-1.92, -0.88) | **<.001** |
| Center effect | Center A (Reference) | | | |
|  | Center B | 0.40 | -0.35 (-1.13, 0.42) | .37 |
|  | Center C | 0.33 | -0.60 (-1.25, 0.04) | .07 |
|  | Center D | 0.30 | -0.72 (-1.31, -0.14) | **.02** |
|  | Center E | 0.25 | -0.76 (-1.25, -0.25) | **.003** |
| Group*Time effect | Control (Reference) | | | |
|  | Intervention*T1 | 0.53 | -0.92 (-1.95, 0.12) | .08 |
|  | Intervention*T2 | 0.53 | -0.36 (-1.41, 0.68) | .50 |

Note. ^a^A age, history of vaginal delivery, history of abortion, mode of delivery, perineal injury, neonatal birth weight, urinary incontinence at baseline and baseline score as adjusted covariates; ^b^UI：Urinary Incontinence; ^c^A Higher score indicates a worse outcome; ^d^Baseline; ^e^Immediately after 12 weeks of intervention; ^f^Six to eight weeks postpartum.
